# Comprehensive of N1-Methyladenosine Modifications Patterns and Immunological Characteristics in Ovarian Cancer

**DOI:** 10.3389/fimmu.2021.746647

**Published:** 2021-10-29

**Authors:** Jinhui Liu, Can Chen, Yichun Wang, Cheng Qian, Junting Wei, Yan Xing, Jianling Bai

**Affiliations:** ^1^Department of Gynecology, The First Affiliated Hospital of Nanjing Medical University, Nanjing, China; ^2^Department of Laboratory Medicine, The First Affiliated Hospital, Nanjing Medical University, Nanjing, China; ^3^Department of Urology, The First Affiliated Hospital of Nanjing Medical University, Nanjing, China; ^4^The Second Clinical School of Nanjing Medical University, Nanjing, China; ^5^Department of Biostatistics, School of Public Heath, Nanjing Medical University, Nanjing, China

**Keywords:** ovarian cancer, m1A modification, tumor microenvironment, prognosis, immune checkpoint blockade

## Abstract

**Background:**

recently, many researches have concentrated on the relevance between N1-methyladenosine (m1A) methylation modifications and tumor progression and prognosis. However, it remains unknown whether m1A modification has an effect in the prognosis of ovarian cancer (OC) and its immune infiltration.

**Methods:**

Based on 10 m1A modulators, we comprehensively assessed m1A modification patterns in 474 OC patients and linked them to TME immune infiltration characteristics. m1Ascore computed with principal component analysis algorithm was applied to quantify m1A modification pattern in OC patients. m1A regulators protein and mRNA expression were respectively obtained by HPA website and RT-PCR in clinical OC and normal samples.

**Results:**

We finally identified three different m1A modification patterns. The immune infiltration features of these m1A modification patterns correspond to three tumor immune phenotypes, including immune-desert, immune-inflamed and immune-excluded phenotypes. The results demonstrate individual tumor m1A modification patterns can predict patient survival, stage and grade. The m1Ascore was calculated to quantify individual OC patient’s m1A modification pattern. A high m1Ascore is usually accompanied by a better survival advantage and a lower mutational load. Research on m1Ascore in the treatment of OC patients showed that patients with high m1Ascore showed marked therapeutic benefits and clinical outcomes in terms of chemotherapy and immunotherapy. Lastly, we obtained four small molecule drugs that may potentially ameliorate prognosis.

**Conclusion:**

This research demonstrates that m1A methylation modification makes an essential function in the prognosis of OC and in shaping the immune microenvironment. Comprehensive evaluation of m1A modifications improves our knowledge of immune infiltration profile and provides a more efficient individualized immunotherapy strategy for OC patients.

## Introduction

Most scientists have focused on the critical effect on RNA methylation modifications in regulating genetic function. Different from DNA methylation, RNA methylation modifications, including N6-methyladenosine (m6A), 5-methylcytosine (m5C) and N1-methyladenosine (m1A), mainly regulate genetic expression at the post-transcriptional level (1–4). Among them, m1A methylation modification refers to inserting a methyl ester to its nitrogen atom at the adenine 1 position of RNA molecules such as mRNA, tRNA, and rRNA (5, 6), and m1A methylation modification is mainly enriched in mRNA 5’-untranslated region (UTR), which is different from most common m6A RNA modification (5, 7). m1A methylation modification maintains the structure and function of non-coding RNAs (ncRNAs), a process that is dynamically reversible and involves three classes of molecules: methyltransferases, demethylases and binding proteins (8). TRMT10C, Trmt61B and TRMT6/61A (methyltransferases) mediate the methylation modification process, as TRMT10C catalyzes m1A at site 9, Whereas the remaining two were catalyzed at site 58 (9–11). Demethylases including ALKBH1 and ALKBH3 can scrub the methylation modification signal from single-stranded DNA and RNA (12–15). The m1A binding proteins comprising YTHDF1, YTHDF2, YTHDF3 and YTHDC1 can read m1A methylation modification information and recognize and bind m1A methylation sites (16). These regulatory genes make an essential function in the process of modifying m1A. More researches have shown that abnormal expression or mutations of m1A regulatory molecules can affect transcription and translation processes, leading to abnormal pathological processes such as abnormal cell proliferation, retarded organismal development and tumorigenesis (17–20).

Using immune checkpoint inhibitors (ICIs), particularly PD-1, PD-L1 and CTLA-4 have become pivotal drugs in tumor-targeted molecular therapy with a brighter therapeutic future (21, 22). Ovarian cancer (OC) is a common malignant carcinoma of female reproductive organs, which seriously threatens women’s lives. Currently, some scholars believe that the application of ICIs can restore T cell function and achieve therapeutic effects (23). However, in clinical practice, most OC patients show resistance to ICIs or the clinical benefits are not as expected (24). The application of ICIs therapy in OC is still controversial (25, 26). Positive response to immunotherapy usually depends on tumor cell interactions (27, 28) and immune regulation within tumor microenvironment (TME) (29, 30). TME consists of tumor cells, neighboring cells, vasculature and the extracellular matrix (ECM). The interaction between tumor cells and other components of TME can induce physiological or pathological changes (31). For example, the immunosuppressive microenvironment in epithelial ovarian cancer (EOC) makes it ineffective for immunotherapy. However, the use of 5-azacytidine has been clinically found to not only increase CD8^+^ T cells and NK cells, but also decrease the macrophage and myeloid suppressor cell in TME. At this point, the tumor immunosuppressive microenvironment is weakened and the efficacy of immune checkpoint therapy is improved (32). Therefore, understanding the interactions and detailed mechanisms between immunotherapy and TME is a priority to improve the efficacy of immunotherapy.

Recently, some researches have concentrated on exploring the relationship between m1A regulators and TME. For example, woo et al. believe that the cytokine CSF-1 has an adverse effect on the prognosis of OC, and m1A methylation is engaged in the degradation of CSF-1 mRNA. Among them, ALKBH3 overexpression increase the stability of CSF-1 and increase CSF-1 expression (18). Wang et al. found that the silencing of TRMT10C suppresses OC proliferation and migration (33). However, the influence of m1A regulators on the development and progression of OC depends on the interaction of multiple regulators, rather than the influence of a single molecule. Thence, a complete assessment of the immune status mediated by multiple m1A regulators will facilitate our insight into the regulatory role of m1A regulators in OC TME.

In this research, we compiled the genetic and clinical information of 474 OC patients to synthetically assess m1A modification modes and correlate them with TME. Ultimately, we identified three m1A modification modes and observed different immune status and prognosis among the modes, which indicates that m1A modification in OC patients makes a critical function in establishing a single TME. Therefore, we developed a scoring system on the basis of genetic profile of the m1A regulators to quantify the m1A modification pattern of each OV patient.

## Methods

### Ovarian Cancer Data Source and Preprocessing

Public RNA-seq expressed data and full clinical annotations are available from The Cancer Genome Atlas (TCGA) and Gene Expression Omnibus (GEO) databases. The exclusion criteria include removing all samples without clinical follow-up information, removing all samples with unknown survival time < 30 days and removing all samples without survival status. After excluding samples with incomplete survival data, a total of 3 eligible OC cohorts, including GSE9891, GSE29691 and TCGA-OV, were collected to be processed. RNA sequencing data of gene expression (FPKM values) and clinical information in TCGA dataset were accessed directly from GDC website (https://portal.gdc.cancer.gov/). FPKM data were converted to transcripts per kilobase million (TPM) data. Normalized RNA sequencing data and corresponded clinical information for GSE9891, GSE29691 microarray dataset were accessed directly from GEO website (http://www.ncbi.nlm.nih.gov/geo). Batch effects between these cohorts were removed using “sva” R package (34). OC patients with mutational data were obtained from TCGA database. A total of 16 OC specimens and 16 normal tissues were obtained from the first affiliated hospital of Nanjing medical university. We obtain all the written informed consent from patients.

### Unsupervised Clustering Analysis of 10 m1A Regulators

Total 10 regulators were selected from TCGA and GEO datasets for determining m1A modification patterns. On the basis of expression of 10 m1A regulators, we used the ConsensuClusterPlus package to apply unsupervised clustering analysis in order to facilitate the identification of different m1A modification patterns and the classification of patients. The consensus clustering algorithm determines how many clusters there are, and the process is repeated a thousand times to ensure reliability (35, 36).

### Gene Set Variation Analysis (GSVA)

GSVA, a non-parametric unsupervised method, can be applied to assess the difference gene set enrichment between different m1A modification modes (37). Download the gene set “c2.cp.kegg.v6.2.symbols” from MSigDB database and use it for running GSVA. An adjusted P<0.05 was regarded as statistically significant.

### TME Immune Cell Infiltration Analysis

CIBERSORT provides expression data for 22 common immune cells LM22. Based on these data, we calculated the association between m1Ascore and immune cell infiltration (38, 39). We utilized the ssGSEA (single sample gene set enrichment analysis) algorithm to assess the degree on immune infiltration. ESTIMATE algorithm was utilized to score stromal and immune gene sets and calculate tumor purity (40).

### Identify Differentially Expressed Genes (DEGs) Between Different Phenotypes of m1A

We applied the empirical Bayesian approach of the limma package to find out DEGs of the three m1A modification patterns (41). The adjusted p for these genes is less than 0.05.

### Construction of m1A Gene Signature

In order to quantify m1A modification pattern of single OC patients, we built a score scheme to assess m1A modification patterns, which we termed as m1Ascore. The m1Ascore was built in the following steps: first, we normalized the DEGs extracted from the different m1Aclusters and extracted overlapping DEGs. overlapping DEGs were analyzed using unsupervised clustering to classify OC patients into several groups. Consensus clustering algorithms were used to determine the count of gene clusters and their stabilization. We conducted prognostic analysis for each overlapping DEG with Cox regression method and screened genes with P<0.05. Principal component analysis (PCA) was used for building m1A-associated gene signature. Principal components 1 and 2 were both chosen for feature scores. m1Ascore = ∑ (PC1i + PC2i). Where i is m1A phenotype-related genes’ expression (42, 43).

### IPS Analysis

IPS is a representative gene associated with immunogenicity calculated using z-score. It uses PD-L1 expression of the four tumor-associated immune cells individually as an evaluation metric to distinguish the beneficiary population. Higher scores were linked to high immunogenicity (44). OC patients’ IPS comes from the Cancer Immunome Atlas (TCIA) (https://tcia.at/home).

### Connectivity Map (CMap) Dataset

CMap, as a gene expression profile database, allows the comparison of differentially expressed gene profiles with database reference datasets to obtain highly correlated agents with diseases (45). We utilized the CMap database to obtain the linkage between m1Ascore, OC and drugs. The 3D structures of the obtained agents can be accessed from Pubchem website.

### External Validation of 10 m1A Regulators Protein Levels

The Human Protein Atlas (HPA) (https://www.proteinatlas.org) includes tissue and cellular protein distribution data from 44 different normal tissue categories and 17 major cancer categories. Immunohistochemical staining intensity, number, location and patient information are available online. After exploring 10 m1A-related genes in HPA database, seven m1A regulators expression (ALKBH1, ALKBH3, TRMT6, TRMT10C, TRMT61B, YTHDC1 and YTHDF2) in normal and OC tissues was obtained.

### Quantitative Real Time PCR

Trizol reagent (Thermo Fisher Scientific, USA) was utilized to isolate and extract RNA from tissue samples. NanoDrop 2000 spectrophotometer (Thermo Scientific, USA) was designed to assess RNA quantity control and concentration. A high-capacity reverse transcription kit (Takara, Japan) was designed for reverse transcription of total RNA to cDNA. qRT-PCR was conducted in a Light Cycler 480II (Roche) using SYBR Green technology (Takara). Record the cycling threshold (Ct) for each gene and calculate the target gene mRNA expression with the 2-ΔΔCt method. All steps of the qRT PCR were performed according to the reagent instructions and all experiments were repeated 3 times. PCR primers are showed in **Table S1**.

### Statistical Analysis

Correlation coefficients between immune cells and m1A regulators expression were calculated by Spearman correlation analysis. Differences between three groups were compared with Kruskal-Wallis test, and associations among categorical covariates were tested with χ2 test (46). Based on the relevance of m1Ascore to patient prognosis, the optimal cut-off value for each dataset sub-group was defined using the survminer R package. This value dichotomizes the patients into high and low m1Ascore subgroups. Log-rank statistics were used for reducing the batch effect of calculations. Kaplan-Meier method was applied for drawing OS plots and log-rank test was utilized to identify statistics differences. Univariate Cox regression were used to compute risk ratios for m1A regulators and genes associated with m1A phenotype. Multivariate Cox regression was applied for identifying independent survival factors as well as the “forestplot” R package to visualize the results. The Maftools package and its “oncoplot” feature are used to present mutational differences. P < 0.05 is considered statistically significant. All data were processed in R 3.6.1 software.

## Results

### m1A Regulators Mutation and Expression Difference and Its Clinical Relevance in OC

**Figure 1:**
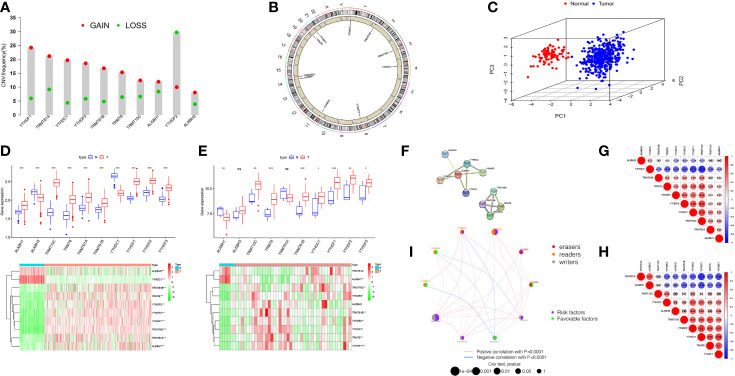
Landscape of genetic and expression variation of m1A regulators in ovarian cancer. **(A)** The CNV variation frequency of m1A regulators in TCGA-OV cohort. The deletion frequency, green dot; The amplification frequency, red dot. **(B)** The location of CNV alteration of m1A regulators on 23 chromosomes using TCGA cohort. **(C)** Principal component analysis for the expression profiles of 10 m1A regulators to distinguish tumors from normal samples in TCGA cohort. **(D, E)** Boxplot and differential expression heatmap of m1A RNA methylation regulators in OC and normal tissues from **(D)** TCGA and **(E)** GEO datasets. **(F)** The PPI network of m1A RNA methylation regulators. **(G)** Spearman correlation analysis of m1A RNA methylation regulators in TCGA cohort. **(H)** Spearman correlation analysis of m1A RNA methylation regulators in GSE27651 cohort. **(I)** The interaction among m1A regulators in ovarian cancer. Violet dots in the circle, risk factors of prognosis; green dots in the circle, favorable factors of prognosis. The lines linking regulators showed their interactions, and thickness showed the correlation strength between regulators. *P < 0.05; **P < 0.01; ***P < 0.001. ns, not significant.

The total workflow is as shown in the following figure (**Supplementary Figure 1**). According to previous literature reports, we identified 10 m1A methylated genes. First, we integrated somatic mutations and copy number variants (CNVs) of these genes to characterize the mutations. As can be seen in **Supplementary Figure 2**, the mean mutation rate of m1A regulators is very low, and only 7 of 436 specimens have m1A regulator mutations, with a frequency of 1.61%. Subsequently, we analyzed the change frequency of the CNV in m1A regulators. **Figure 1A** shows that copy number variation was present in all 10 regulators, of which, most were dominated by copy number amplification, with only YTHDF2 exhibiting a copy number deletion. **Figure 1B** further visualizes the location of CNV alterations in the m1A regulators on the chromosome. By analyzing the expression of these 10 m1A regulators, we identified two subgroups that did not cross over, which demonstrated that we could completely distinguish between OC and normal samples based on m1A regulators expression levels (**Figure 1C**).

For searching the link between m1A methylation regulators and OC, we compared m1A regulator mRNA expression in OC and normal tissues from two databases, TCGA and GEO, respectively. TCGA data showed aberrant expression of all m1A regulators in OC. The expression of all genes in OC was greater than that in normal (p<0.001, **Figure 1D**). However, the different expression of these m1A regulators was not the same as that in GSE27651 database. In GSE27651 dataset, there was no statistical difference in ALKBH3 and TRMT61A expression between OC and normal samples. And compared with normal tissues, ALKBH1 expression (P<0.01) was significantly decreased in OC, while the expression of YTHDC1 increased (P<0.05, **Figure 1E**). Furthermore, we compared the differences in m1A regulator expression at the protein level between two tissues. The results proved that ALKBH3 expression was lower in tumor compared to normal tissues, TRMT61A expression differences were not meaningful and the rest were expressed higher in OC (**Supplementary Figure 3A**). The above analysis indicates a high degree of heterogeneity in genetic variation and expression differences between normal and OC tissues, which indicates that the imbalance in the expression of m1A regulators makes a critical function in the development of OC and that genetic variation may interfere with their expression levels.

Next, we tried to clarify the relationship among the 10 m1A RNA methylation regulators. Analysis of the String database (**Figure 1F**) showed that ALKBH1 may be a central gene of the m1A RNA methylation regulator. However, further analysis did not display an expression correlation between ALKBH1 and other regulators. Interestingly, in TCGA cohort, we found that TRMT10C was positively related with seven m1A regulators, particularly YTHDF1 and YTHDF2. In contrast, YTHDC1 was negatively correlated with other eight regulators, particularly YTHDF1 and TRMT10C (**Figure 1G**). This suggests that TRMT10C and YTHDC1 may be key genes in m1A RNA methylation regulators affecting OC occurrence and development. However, in the GSE27691 cohort, we found that TRMT61A was negatively correlated with the expression of seven m1A regulators, particularly YTHDC1 and YTHDF1. In contrast, YTHDF1 was positively correlated with six other regulators, particularly YTHDC1 (**Figure 1H**). Broadly speaking, the correlation trends between the regulators in the TCGA and GEO databases were generally consistent.

We discuss the prognostic relevance of m1A regulators in patients with OC (**Supplementary Figure 3B**). We found that some m1A regulators, including ALKBH1, ALKBH3, TRMT6, TRMT10C, YTHDF1 and YTHDF2 are oncogenic and that overexpression of these genes results in worse prognosis for OC patients. The m1A regulator network diagram further describes the connections and interactions between m1A regulators and their prognostic significance for OV patients (**Figure 1I**). We revealed that m1A regulators expression showed significant correlations both within the same category and between different categories.

### m1A Regulators-Mediated m1A Modification Patterns

**Figure 2:**
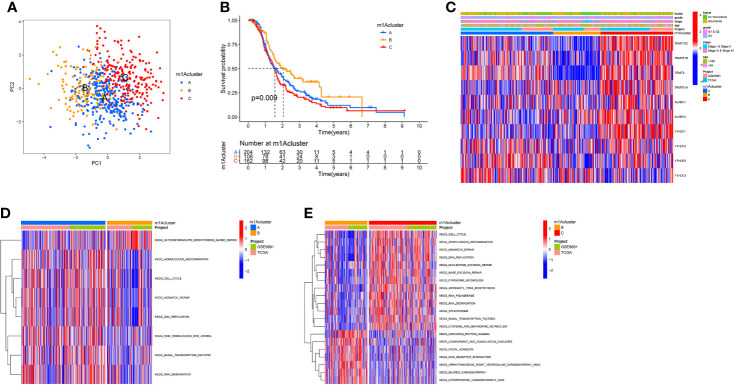
The m1A modification patterns in OC and biological characteristics of m1A subtypes. **(A)** The principal component analysis for the transcriptome profiles of three m1A modification patterns. **(B)** Survival analyses of the three m1A modification patterns based on 611 OC patients. **(C)** Unsupervised clustering of 10 m1A regulators in two independent ovarian cancer cohorts. **(D, E)** GSVA enrichment analysis showing the activation states of biological pathways in distinct m1A modification patterns. The red represented activated pathways and blue represented inhibited pathways. **(D)** The m1A cluster A *vs* m1A cluster B; **(E)** The m1A cluster B *vs* m1A cluster C.

According to 10 m1A regulator expression, we used R program to classify OC patients with different m1A modification patterns and eventually identified three different modification patterns by using an unsupervised clustering approach, namely m1A cluster-A (227 patients), B (148 patients), and C (236 patients) (**Figure 2A** and **Supplementary Figures 4A–C**). To discover the prognostic worth of the three m1A modification patterns, we concluded Kaplan-Meier analysis, the survival curves showed that the m1A cluster-B modification pattern was more pronounced among the three m1A modification subtypes (P = 0.009, **Figure 2B**). We also plotted heat maps to discuss the correlation between clinicopathological characteristics of OC patients and m1A modification patterns (**Figure 2C**).

Subsequently, we performed GSVA enrichment analysis of the three m1A modification patterns to investigate the associated pathways and biological significance. As shown in **Figures 2D, E**, m1A cluster-A and m1A cluster-C are similar in that both are significantly enriched for a number of gene replication and repair-related pathways, including homologous recombination, mismatch repair, and DNA replication. Differently, ECM-receptor interaction and focal adhesion were heavily abundant in m1Acluster-B, which may be related to the cellular matrix and its role in TME.

### Immune Characteristics in Different m1A Modification Patterns

**Figure 3:**
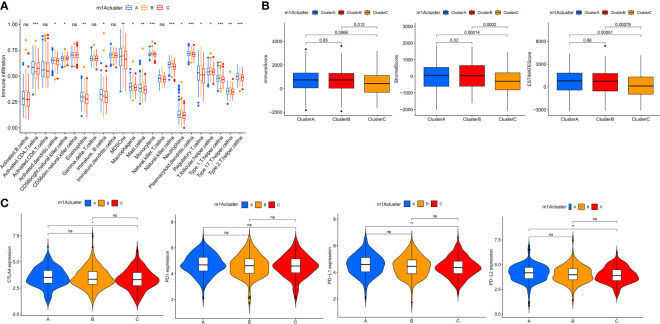
TME cell infiltration characteristics and immune checkpoints in distinct m1A modification patterns. **(A)** The abundance of each TME infiltrating cell in three m1A modification patterns. **(B)** The box plot indicated the correlation between different m1A modification patterns and immune scores, stromal scores and estimate score. **(C)** The expression of CTLA-4, PD1, PD-L1 and PD-L2 among distinct m1A modification patterns. *P < 0.05; **P < 0.01; ***P < 0.001. ns, not significant.

To better understand the relationship between m1A modification patterns and immunity, we explored immune characteristics of different m1A modification patterns. Analysis of cellular infiltration in the TME showed that the three m1A modification patterns had distinctly different TME cellular infiltration characteristics, with the most abundant immune cellular infiltration in m1A cluster-B, including many CD4+ T cells, plasmacytoid dendritic cells, monocyte, NK cells, MDSC, etc. (**Figure 3A**). This immunological profile corresponds to a tumor immune-inflamed phenotype (47), and tumor patients with this profile are the most responsive to immunotherapy, and m1A cluster-B patients do show a matched survival advantage (**Figure 2B**).

Furthermore, the results of GSVA analysis showed (**Figure 2E**) that the cluster B modification pattern significantly correlated with intrastromal interactions. Therefore, we hypothesized that the matrix in cluster B activates the anti-tumor effects of immune cells. We then employed ESTIMATE algorithm to calculate the proportion of the immune matrix component of each m1A modification pattern in the TME and presented it as immune score, stromal score and estimate score, which were positively correlated with immunity, stroma and the sum of both. We found that clusters-A and B had significantly more immune and stromal components in TME than cluster-C (p<0.05, **Figure 3B**).

In clinical tumor immunotherapy, the main immune checkpoint inhibitors currently in use include CTLA-4 inhibitors and PD-1 inhibitors. In order to advance the study of ICIs, understanding immune checkpoints expression in three m1A modification patterns is necessary. Therefore, we explored immune checkpoints expression in different m1A modification patterns. As seen in **Figure 3C**, immune checkpoint expression was slightly different in m1A subtypes, with slightly higher immune checkpoint expression in cluster A compared to cluster C (p < 0.05).

### Construction of m1A Gene Signature and Its Immunological and Clinical Characterization

**Figure 4:**
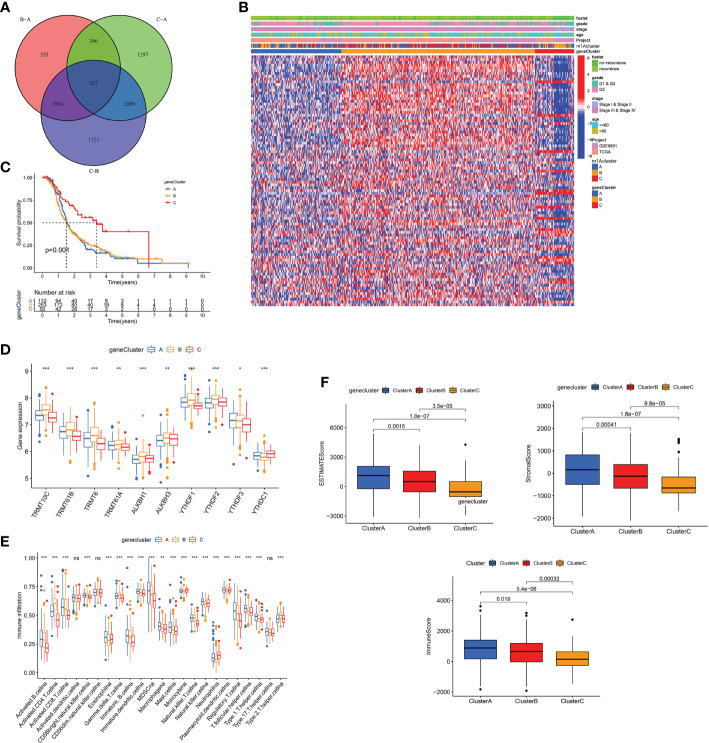
The m1A gene clusters in OC and biological characteristics of m1A gene subtypes. **(A)** 527 m1A phenotype-related genes shown in venn diagram. **(B)** Unsupervised clustering of overlapping m1A phenotype-related genes in TCGA and GEO cohorts to classify patients into different genomic subtypes, termed as m1A gene cluster A-C, respectively. The gene clusters, m1A clusters, tumor stage, grade, survival status and age were used as patient annotations. **(C)** Kaplan-Meier curves indicated m1A modification genomic phenotypes were markedly related to overall survival of OC patients. **(D)** The expression of 10 m1A regulators in three m1A gene clusters. **(E)** The abundance of each TME infiltrating cell in three m1A gene clusters. **(F)** The box plot indicated difference in immune scores, stromal scores and estimate score between three m1A gene clusters. *P < 0.05; **P < 0.01; ***P < 0.001. ns, not significant.

To explore the biology of each m1A modification pattern, we identified 527 m1A-associated DEGs (**Figure 4A**) and conducted GO and KEGG analysis on these genes (**Supplementary Figure 4D**). Based on 527 m1A phenotype-related genes, an unsupervised cluster analysis was then performed, and the patients were divided into different genomic groups. Similarly, unsupervised clustering algorithm exhibited three m1A modification genomic phenotypes, which termed as m1A gene clusters A, B, C (**Figure 4B** and **Supplementary Figures 4E–G**). This demonstrates that there are indeed three distinct patterns of m1A modification in OC. We observed that the three different gene clusters have different clinical characteristics (**Figure 4B**). Compared with m1A gene cluster-A and cluster-C, OC patients in m1A gene cluster-B displayed poor differentiation and higher frequency of recurrence (**Figure 4B**); and of the three m1A gene clusters, patients in gene cluster-C had longer survival times (p=0.001, **Figure 4C**). Moreover, m1A regulators’ expression in the three m1A gene clusters also had significant differences (**Figure 4D**). In addition, we performed immune correlation analysis on the m1A gene cluster A-C. As shown in **Figure 4E**, the three m1A gene modification patterns have different TME cell infiltration characteristics. Unlike the m1A modification pattern, the m1A gene modification pattern has the most abundant immune cell infiltration in m1A gene cluster-A, such as activated CD8+T cell, MDSC, immature DC, monocyte, plasmacytoid DC, etc. After applying ESTIMATE algorithm, we found that gene cluster-A has the most immune and matrix components in TME, followed by gene cluster-B (P<0.05, **Figure 4F**). These findings demonstrate that m1A methylation modifications play an important regulatory effect on building of different TME landscapes.

**Figure 5:**
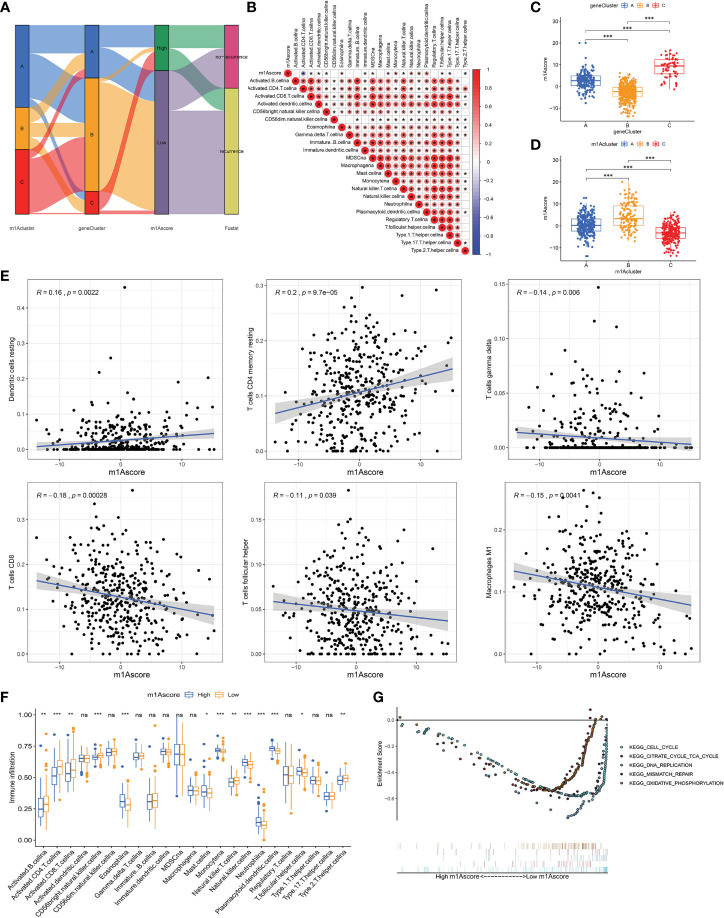
Construction of m1A signatures. **(A)** Alluvial diagram showing the changes of m1Aclusters, gene cluster, m1Ascore and patient survival status. **(B)** Correlations between m1Ascore and the known gene signatures using Spearman analysis. Negative correlation was marked with blue and positive correlation with red. **(C)** Differences in m1Ascore among three gene clusters. **(D)** Differences in m1Ascore among three m1A modification patterns. **(E)** The correlation between m1Ascore and several immune cell, including resting dendritic cells, macrophages M1, resting memory CD4+ T cells, CD8+ T cells, follicular helper T cells and gamma delta T cells. **(F)** The abundance of each TME infiltrating cell in low- and high-m1Ascore group. **(G)** Enrichment plots showing cell cycle, citrate cycle TCA cycle, DNA replication, mismatch repair, and oxidative phosphorylation pathways were enriched in the low m1Ascore subgroup. *P < 0.05; **P < 0.01; ***P < 0.001. ns, not significant.

Due to individual differences in m1A modification, based on 527 DEGs, we built a score system to accurately assess m1A modification pattern of single OC patients, and termed it the m1Ascore. First, the alluvial map visually displays the attribute changes of a single OC patient (**Figure 5A**). To better characterize the immunological profile of m1A feature, we analyzed the association between immune cells and m1Ascore (**Figure 5B**). Kruskal-Wallis test showed significant differences in m1Ascore between m1A gene clusters. Gene cluster-C scored highest median value and the lowest was for gene cluster-B (**Figure 5C**). In contrast, the highest median value was observed in m1A cluster B, while the lowest median value was observed in m1A cluster C (**Figure 5D**). Combining previous survival analysis in different clusters (**Figures 2B** and **4C**), this indicates that the m1Ascore may be positively correlated with the survival of OC patients.

Until we determined the prognostic value of m1Ascore, we evaluated the significance of the m1Ascore in immunological terms. First, we examined the correlation between immune cells and m1Ascore, and found that M1 macrophages, CD8^+^ T cells, gamma delta T cells, T follicular helper cells decreased as m1Ascore increased, with a negative correlation between the two; in contrast DCs and resting CD4 memory T cells were positively related to m1Ascore, and cell infiltration increased with increasing score (**Figure 5E**). Subsequently, following the optimal cutoff values defined from survminer package, we dichotomized OC specimens into high- and low-m1Ascore groups and initially assessed the TME immune infiltration in these two groups. **Figure 5F** shows that intrinsic immune cells, including monocyte, NK cells and plasmacytoid DCs, were predominantly increased in high m1Ascore subgroup. On the contrary, there are more adaptive immune cells, consisting of B cells, CD4+ T cells and CD8+ T cells in low m1Ascore subgroup. Similarly, we apply the ESTIMATE algorithm between the two groups, and noticed no significant difference in infiltrating components in TME between the two groups (**Supplementary Figure 4H**). Furthermore, GSEA revealed that the low m1Ascore group mainly enriched some metabolic pathways and gene replication and repair-related pathways, including DNA replication and oxidative phosphorylation signaling. These are all tumor-related pathways, suggesting a poor prognosis for the low m1Ascore group (**Figure 5G**).

**Figure 6:**
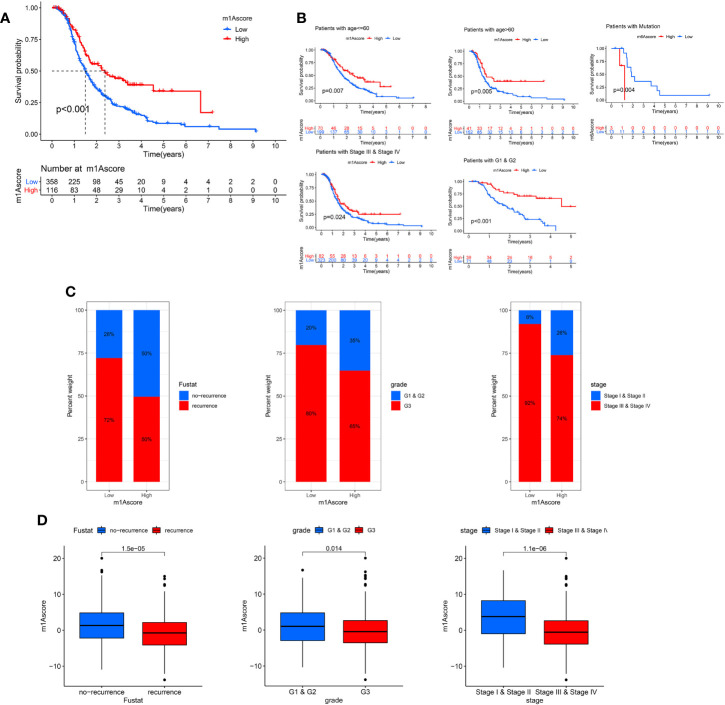
Characteristics of m1A modification in OC patient subtypes. **(A)** Survival analyses of low and high m1Ascore patient groups in OC cohort using Kaplan-Meier curves. **(B)** Kaplan-Meier curves depicted the survival difference between low and high m1Ascore in the stratified analysis of OC patients, including age, grade, stage, and BRCA mutation. **(C)** The proportion of patient survival status, stage and grade in high and low m1Ascore groups. **(D)** Boxplots for m1Ascore between different characteristics OC patients, including patient survival status, stage and grade.

Next, we deeply analyze the value of m1Ascore in the prognosis of OC patients. High m1Ascore group patients showed better survival benefits (**Figure 6A**). Similarly, the high m1Ascore group consistently showed a marked survival advantage in patients stratified by different clinical characteristics (**Figure 6B**). Moreover, high m1Ascore group had a significantly greater proportion of early-stage patients and recurrence-free population (**Figure 6C**), and these patients had higher m1Ascore (P<0.05, **Figure 6D**). This means that high scoring populations characterized by m1Acluster-B modification modes and immune activation phenotypes have a more favorable prognostic outlook. The above outcomes indicate that m1Ascore can also be employed to assess several clinical features of OC patients, including clinical stage, grade, and survival status.

### Correlation Between m1A Score and Tumor Burden Mutation

**Figure 7:**
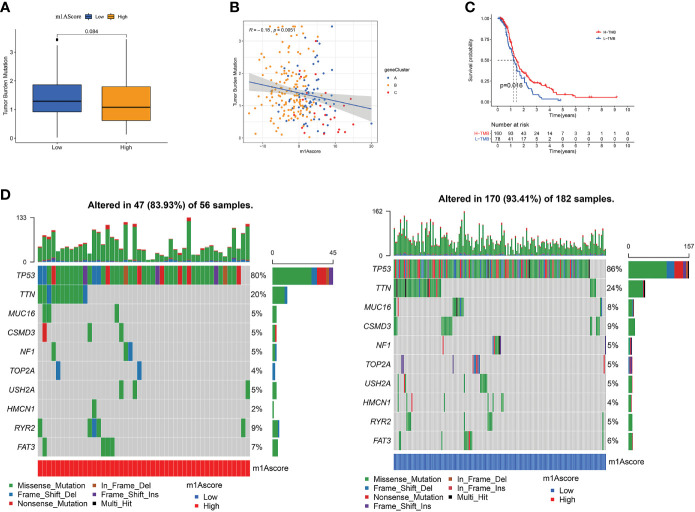
The Correlation between the m1Ascore and somatic variants. **(A)** TMB difference in the high and low m1Ascore subgroups. **(B)** Scatterplots depicting the positive correlation between m1Ascores and tumor mutation load. **(C)** Kaplan-Meier curves for high and low TMB groups of the TCGA-OV patient. **(D)** The single-nucleotide variant was constructed using high m1Ascores on the left (red) and low m1Ascores on the right (blue). Individual patients are represented in each column.

There is a statistics association between tumor burden mutations (TMB) and tumor stage, grade, residual tumor size, and immune infiltrating cells in TME, which indicates that TMB has a major role in predicting OC survival and guiding immunotherapy in OC patients (48). Given TMB’s clinically important nature, we attempted to probe the inherent relevance between TMB and m1Ascore to clarify genuine markings of the two groups. First, as shown in **Figure 7A**, although not statistically significant, low m1Ascore subgroup patients showed greater TMB than high m1Ascore group. Correlation analysis also confirmed that m1Ascore is significantly negatively associated with TMB (Spearman coefficient: R = -0.18, p = 0.0051; **Figure 7B**). Then, patients were dichotomized into high and low TMB groups. As shown in **Figure 7C**, we observed that OS was better in high-TMB patients (p=0.016). Collectively, these findings indicate that m1Ascore can be treated a predictor independent of TMB. Besides, we use maftools package to compare the distribution of somatic variation between low and high m1Ascore groups in TCGA. **Figure 7D** illustrates that low m1Ascore group exhibited a more extensive TMB than the high m1Ascore subgroup. These results provide new insights into the mechanisms underlying tumor m1Ascore composition and gene mutation, and indirectly confirm the value of m1Ascore in predicting the outcome of immunotherapy

### The Role of m1A Modification Patterns in Ovarian Cancer Treatment

**Figure 8:**
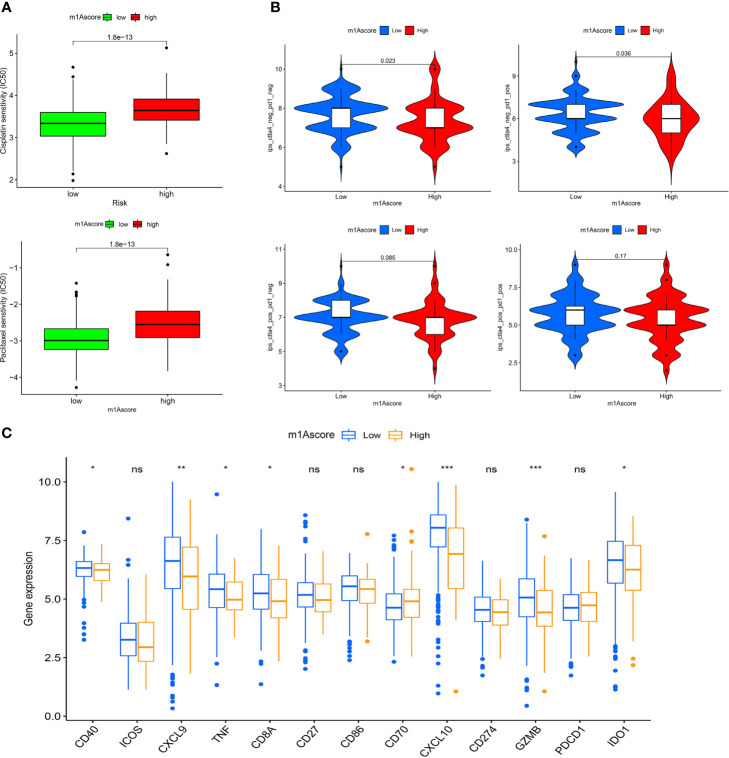
m1A modification patterns in the role of OC clinical therapies **(A)** Box plot showing the sensitivity of patients with high and low m1Ascore subgroups to chemotherapy drugs, including cisplatin and paclitaxel. **(B)** The association between IPS and immune checkpoints in OC patients with different m1Asocre. **(C)** The expression of immune-relevant genes in high and low m1Ascore subgroups. *P < 0.05; **P < 0.01; ***P < 0.001; ns, not significant.

In recent years, platinum-based drugs and paclitaxel have been used as representative drugs for chemotherapy of OC (49). For this reason, based on the two treatment cohorts of TCGA and GSE9891, we explored whether m1A modification characteristics can predict the response of patients to these two first-line chemotherapeutic agents, including cisplatin and paclitaxel. It can be seen from **Figure 8A** that patients with low m1Ascore showed significant treatment sensitivity to both groups of drugs, which indicates that the m1Ascore facilitates the forecasting of patient reaction to chemotherapy.

**Figure 9:**
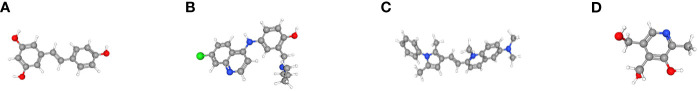
The 3D structure of the four small molecule drugs for OC. **(A)** resveratrol, **(B)** amodiaquine, **(C)** pyrvinium, and **(D)** pyridoxine.

Moreover, we utilized CMap database to examine the potential role of m1Ascore in the development of new drugs for OC. First, we screened for differentially expressed genes between the groups with high and low m1Ascore (**Supplementary Figures 5A, B**) and conducted GO and KEGG enrichment analysis on these genes (**Supplementary Figures 5C, D**). Subsequently, we utilized CMap database to analyze these genes and find drugs with high association with OC. The top 10 small molecule drugs highly correlated with OC are shown in **Table 1**. Among these agents, we obtained chemical structure information from PubChem for 4 most important small molecule agents (**Figures 9A–D**).

**Table 1:** Results of CMap analysis.

cmap name	mean	n	enrichment	p-value	specificity	percent non-null
resveratrol	0.520	9	0.700	0.00006	0.0931	66
guanabenz	-0.440	5	-0.859	0.00008	0	60
amodiaquine	0.708	4	0.900	0.0001	0	100
indoprofen	-0.673	4	-0.910	0.0001	0	100
oxybenzone	-0.584	4	-0.851	0.00092	0.0141	75
pyrvinium	0.543	6	0.730	0.00103	0.0884	66
pyridoxine	0.386	4	0.836	0.00111	0	50
natamycin	0.367	4	0.808	0.00253	0	50
aciclovir	-0.352	6	-0.679	0.00294	0.0199	50
antimycin A	0.464	5	0.715	0.00439	0.0281	60

ICI has emerged as a key drug for immunotherapy (23). However, there is still several patients who do not respond to immunotherapy, which to some extent limits the application of ICIs. Therefore, Charoentong et al. developed a quantitative scoring scheme called immune phenotype score (IPS) to ascertain the determinants that influence tumor immunogenicity. In this scoring scheme, IPS is an excellent predictor for detecting anti PD-1 and anti CTLA-4 antibody responses (44). Here, we completely analyzed the link that exists between IPS and immune characteristics. The IPS-CTLA4-neg-PD-1-neg, IPS-CTLA4-neg-PD-1-pos, IPS-CTLA4-pos-PD-1-neg, IPS-CTLA4-pos-PD-1-pos scores were designed to assess the likelihood of patients receiving ICIs therapies. The results proved that the score increased significantly in the high m1Ascore group (**Figure 8B**): IPS-CTLA4-neg-PD-1-neg, P = 0.023; IPS-CTLA4-neg-PD-1-pos, P = 0.036. This indicates that the high m1Ascore group shows higher IPS and appears to have more immunogenic phenotypes. These results suggest that the effectiveness of ICIs may be better in patients with high m1Ascore.

Besides, we also investigated some common immune molecules expressions in different m1Ascore groups, including CD274, PDCD1, CD40, CXCL9 and so on. We observed that most genes, except CD70, showed higher expression levels in the low m1Ascore group (**Figure 8C**). This may also be a major factor in the lower survival rate of low m1Ascore patients (**Figure 6A**). These results above imply that quantification of m1A modification patterns might be used as a prospective and stable biomarker for chemotherapeutic response and immunotherapy evaluation.

### Clinical Validation of Proteins, mRNA Expression of m1A Regulators

**Figure 10:**
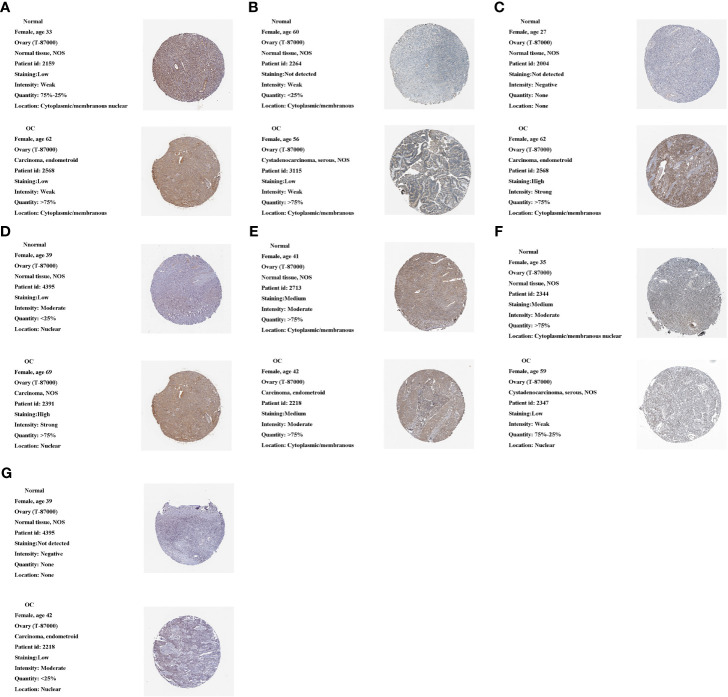
Protein expression of 10 m1A regulators in the tumor and normal tissues in the HPA database. **(A)** ALKBH1 expression. **(B)** ALKBH3 expression. **(C)** TRMT10C expression. **(D)** TRMT6 expression. **(E)** TRMT61B expression. **(F)** YTHDC1 expression. **(G)** YTHDF2 expression.

We used the HPA database to analyze m1A regulator protein expression. Since immunohistochemical data for TRMT61A, YTHDF and YTHDF3 were missing from the HPA data, we only analyzed the remaining seven m1A regulators. Compared to the normal tissue, TRMT10C, TRMT6 and YTHDF2 were relatively highly expressed in the tumor tissue, while YTHDC1 was relatively less expressed. TRMT61B was moderately expressed in both tumor and normal samples. ALKBH1 and ALKBH3 were both lowly expressed in tumor and normal samples (**Figures 10A–G**).

**Figure 11:**
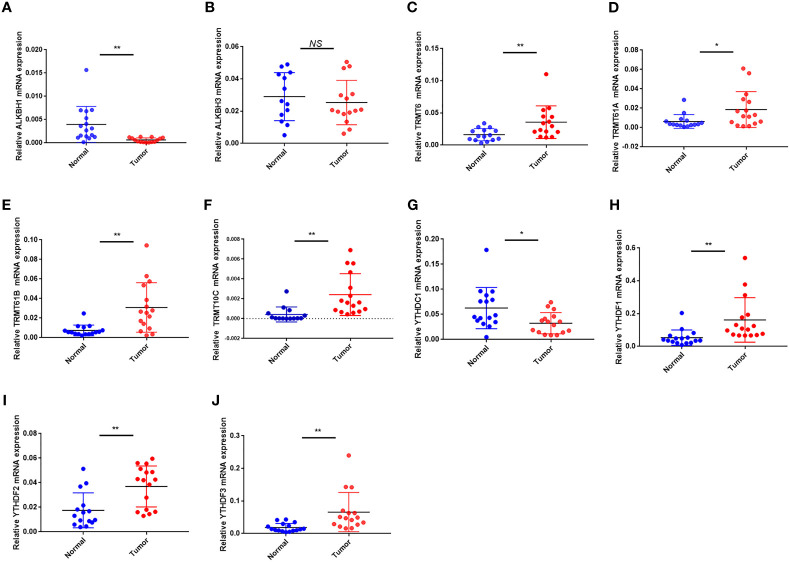
The mRNA expression of the 10 m1A regulators in tumor and normal samples. **(A–J)** The mRNA expression of **(A)** ALKBH1, **(B)** ALKBH3, **(C)** TRMT6, **(D)** TRMT61A, **(E)** TRMT61B, **(F)** TRMT10C, **(G)** YTHDC1, **(H)** YTHDF1, **(I)** YTHDF2 and **(J)** YTHDF3. *P < 0.05; **P < 0.01; ns, not significant.

In terms of mRNA levels, TRMT6, TRMT61A, TRMT61B, TRMT10C, YTHDF1 and YTHDF2 YTHDF3 were overexpressed in tumor sample. While ALKBH1 and YTHDC1 were overexpressed in normal sample. ALKBH3 expression had no difference between the tumor and normal sample (**Figures 11A–J**). The mRNA expression detected by PCR was in general agreement with the TCGA database (**Figure 1D**).

## Discussion

More and more evidences show that under the interaction between multiple m1A regulators, m1A modification makes a critical function in inflammation, immune response, and tumorigenesis (50, 51). Most previous researches have focused on a single cell or a single regulator (51, 52), and thus a comprehensive understanding of the overall infiltration characteristics of the immune microenvironment mediated by the co-regulation of multiple m1A regulators is still lacking. Lately, m6 A modification has been fully explored in the tumor immune infiltration of OC (53). In this research, we highlighted the effect of m1A modifications in TME cell infiltration to enhance the knowledge of anti-tumor immune response and allow for targeted immunotherapy regimens to be proposed.

In our research, on the basis of 10 m1A regulators, we identified three different m1A modification patterns. Each of the three modification patterns has a different TME immune cell infiltration profile, where the ESTIMATE algorithm showed substantial innate immunity and matrix activation in both cluster-A and B. Combined with immune cell infiltration profile, we observed that cluster-B corresponds to immune-inflamed phenotype, which contains many CD4^+^ T cells accompanied by myeloid cells and monocytes infiltration that activates an adaptive immune response. Cluster-A corresponds to the immune-excluded phenotype. Although there is also a large infiltration of immune cells within this tumor phenotype, most immune cells are present in the matrix that surrounds the nests of tumor cells, instead of penetrating the parenchyma. This limits tumor entry for immune cells to exert their immune effects. The characteristics of cluster-C correspond to an immune-desert phenotype. The immune-desert phenotype is related to immune tolerance and absence of T cell activation (47). By combining the immune cell infiltration profiles of individual clusters, this confirms the validity of the immunophenotypic categorization of the various m1A modification patterns. Thus, having thoroughly explored TME cell infiltration profiles induced by different m1A modification patterns, improves the future application of precision-focused, personalized therapy against OC. Furthermore, in this research, differences in the mRNA transcriptome between different m1A modification patterns have been demonstrated to be markedly linked to m1A and cellular matrix-related biological pathways. These DEGs are considered to be m1A-associated signature genes. In agreement with the clustering of m1A-modified phenotypes, three genomic clusters were defined on the basis of DEGs that were likewise associated with matrix and immune activation. This reaffirms important role of m1A modifications in structuring the diverse TME landscape. Hence, a comprehensive evaluation of m1A modification patterns will strengthen our visibility into the infiltration features of TME cells.

Given individual differences in m1A modification, a scoring system was developed to precisely assess the pattern of m1A modification in single OC patients, referred to as the m1Ascore. m1Acluster B, characterized by an immune-inflamed phenotype, showed the highest m1Ascore and the best prognostic outcome, whereas the immune-desert phenotype, characterized by m1Acluster C was the exact opposite. These results were also fully validated in the m1A genomic clusters. This suggests that m1Ascore is a reliable prognostic factor in OC and can be used to comprehensively assess individual tumor m1A modification patterns.

ICTs, including anti-PD-1 and CTLA-4 therapies have completely improved therapeutic and prognostic profile for a variety of advanced cancers, including OC (54). Although the survival of OC patients receiving immunotherapy has been significantly improved, there are obvious individual differences in the response of patients to immunotherapy. Therefore, finding markers that predict the results of immunotherapy is of clinical relevance. Earlier studies indicated that large CD8+ T cell infiltration plus the presence of nonsynonymous mutations propel the reaction to anti-PD-1 therapy. Furthermore, by confirming the predictive worth of m1Ascore in two anti-PD-1 and anti-CTLA-4 immunotherapy cohorts, we discovered that high m1Ascore patients were more likely to be treated with ICIs. Thus, we prove that m1A modification patterns play a nontrivial part in molding distinct immune TME landscapes, meaning that m1A modification influences ICI treatment efficacy.

Luckily, we have found several small molecule medicines, such as resveratrol, amodiaquine, pyrvinium and pyridoxine, that can improve ovarian cancer treatment outcomes. Among them, resveratrol, as a natural polyphenolic organic compound, has antioxidant, anti-inflammatory, anti-cancer and cardiovascular protective functions. In a variety of tumor models, including ovarian cancer and pancreatic cancer, resveratrol was proven to be potent in controlling tumor cell proliferation and cancer development. Nevertheless, resveratrol is impacted by several factors during cancer treatment and further clinical studies are needed to confirm its role (55, 56). Usually, amodiaquine is applied to treat various types of malaria, acting in the intra-erythrocytic phase, mainly controlling symptoms quickly and with good tolerability (57). Currently, some studies have revealed that amodiaquine has potential anticancer effects in some cancers and has a wide range of applications (58, 59). Pyrvinium, as a cyanamide dye-like compound, has a significant anti-pinworm effect, interfering with the respiratory enzyme system of the worm and inhibiting oxygen uptake. In clinical trials, pyrvinium pamoate is considered to be an anthelmintic with anticancer effects. It kills tumor cells and restrains cancer cell metastasis by suppressing lipid anabolic metabolism (60, 61). Pyridoxine, also known as vitamin B6, is an essential trace element for maintaining the metabolic and regulatory processes of the body. Some studies have proven that pyridoxine intake is negatively associated with breast cancer risk, which means that pyridoxine has a potential protective function on the risk of breast cancer (62, 63). The above-mentioned agents have their own different effects in the anti-tumor field. However, clinical trials are still needed to validate the application of these drugs in OC patients.

Finally, we measured mRNA and protein expression levels of m1A regulators in OC and normal samples. We found higher protein and mRNA expression of TRMT6, TRMT10C and YTHDF2 in tumor samples. TRMT61A, TRMT61B, YTHDF1 and YTHDF3 mRNAs were higher in tumor tissues, but protein expression was weaker or not apparently distinct between tumor and normal samples. ALKBH1 and ALKBH3 mRNAs were higher in normal samples, but protein expression did not differ markedly between tumor and normal samples. YTHDC1 protein mRNA and mRNA expression were higher in normal tissues. mRNA and protein expression were broadly in agreement with TCGA and GEO databases.

However, our study also has some deficiencies. Our research materials are all derived from databases, and the results are obtained on the basis of bioinformatics. There is a lack of clinical cohorts and prospective clinical trials to validate the correlation between m1A modification and tumor immunity.

In clinical applications, m1Ascore can be applied to synthetically assess the m1A methylation modification pattern and the corresponding immune cell infiltration characteristics in individual patients, so as to facilitate the determination of tumor immunophenotypes and guide more effective clinical medications. We also proved that m1Ascore can be used not only to assess the clinicopathological characteristics of patients, including clinical stage and tumor mutation burden, but also as an independent prognostic biomarker. Furthermore, we validated that m1Ascore predicts the effectiveness of adjuvant chemotherapy and the clinical response of patients to anti-PD-1/CTLA-4 therapy. Thus, we herein provide clinicians with new ideas for immuno-oncology and individualized immunotherapy in OC.

## Conclusion

In conclusion, this research investigated the regulatory role of m1A methylation modifications in the immune microenvironment of OC. m1A methylation modifications are one of the critical factors contributing to the heterogeneity of individual tumor immune infiltration. A thorough evaluation of m1A modification patterns in single OC patients may facilitates our comprehension of tumor immune landscape and provide more efficacious therapeutic approaches for OC patients.

## Data Availability Statement

The datasets presented in this study can be found in online repositories. The names of the repository/repositories and accession number(s) can be found in the article/**Supplementary Material**.

## Ethics Statement

The studies involving human participants were reviewed and approved by Nanjing Medical University. The patients/participants provided their written informed consent to participate in this study. Written informed consent was obtained from the individual(s) for the publication of any potentially identifiable images or data included in this article.

## Author Contributions

YX and JB conceived the study and participated in the study design, performance, and manuscript writing. JL and CC conducted the bioinformatics analysis. YW, CQ, and JW revised the manuscript. All authors contributed to the article and approved the submitted version.

## Conflict of Interest

The authors declare that the research was conducted in the absence of any commercial or financial relationships that could be construed as a potential conflict of interest.

## Publisher’s Note

All claims expressed in this article are solely those of the authors and do not necessarily represent those of their affiliated organizations, or those of the publisher, the editors and the reviewers. Any product that may be evaluated in this article, or claim that may be made by its manufacturer, is not guaranteed or endorsed by the publisher.

## Supplementary Material

Supplementary Figure 1 The workflow employed in the development of the m1Ascore.

Supplementary Figure 2 The mutation frequency of 10 m1A regulators in 436 patients with ovarian cancer. Each column represented individual patients. The upper barplot showed TMB and the number on the right indicated the mutation frequency in each regulator. The right barplot showed the proportion of each variant type. The stacked barplot below showed fraction of conversions in each sample.

Supplementary Figure 3 **(A)** Differential expression of the m1A methylation regulators at the protein level. **(B)** Kaplan-Meier curves demonstrate the prognostic relevance of m1A methylation regulator expression to OC patients.**(A)**

Supplementary Figure 4 **(A)** Cumulative distribution function curves for unsupervised clustering of 10 m1A regulators, k = 2-9. **(B)** Relative change in area under the CDF curve for unsupervised clustering of 10 m1A regulators, k = 2-9. **(C)** Heat map of the consensus matrix for the OC sample at k = 3. **(D)** Gene Ontology (left) and Kyoto Encyclopedia of Genes and Genomes (right) enrichment analyses of 527 m1A phenotype-related DEGs. **(E)** Cumulative distribution function curves for unsupervised clustering of 527 m1A phenotype-related genes in OC cohort, k = 2-9. **(F)** Relative change in area under the CDF curve for unsupervised clustering of 527 m1A phenotype-associated genes, k = 2-9. **(G)** Heat map of the consensus matrix for k = 3. **(H)** The box plot indicated difference in immune scores, stromal scores and estimate score between low and high m1Ascore subgroup

Supplementary Figure 5 **(A)** Volcano plot of differentially expressed genes between high and low m1Ascore groups. **(B)** heatmap of differentially expressed genes between high and low m1Ascore groups. **(C)** Kyoto Encyclopedia of Genes and Genomes and **(D)** Gene Ontology enrichment analyses of differentially expressed genes between groupings of high and low m1Ascore.
