# Supplementary material for: Comprehensive of N1-Methyladenosine Modifications Patterns and Immunological Characteristics in Ovarian Cancer
Source: Front Immunol. 2021 Oct 29;12:746647. doi: 10.3389/fimmu.2021.746647 (PMC8588846; doi:10.3389/fimmu.2021.746647)
Supplement: Supplementary file 7 [file Table_1.docx]

**Table S1 Primer sequences for 10 m1A regulators**

| **Gene** | **Primer Sequences (5'-3')** |
| --- | --- |
| RG9MTD1-F | TCAAGCTGCTAGAAACCACTG |
| RG9MTD1-R | TCTGTGCAAAGCACCATCTATT |
| TRMT61B-F | TGGATGTCTTTCGTCACTGGA |
| TRMT61B-R | CTGCGAGAGCATCGAAGGAT |
| TRMT6-F | GGTGCTGAAACGTGAAGATGT |
| TRMT6-R | CTTGGGCTGTAGACTTCCTCC |
| TRM61-F | AGCTTCGTGGCATACGAGG |
| TRM61-R | CCGATAAGGTCAACTGAGTGC |
| ALKBH1-F | AGAAGCGACTAAACGGAGACC |
| ALKBH1-R | GGGAAAGGTGTGTAATGATCTGC |
| ALKBH3-F | AGGACCGGCATCAGAGAGG |
| ALKBH3-R | CAATGCGGTTCTTTAGTGTGC |
| YTHDC1-F | AACTGGTTTCTAAGCCACTGAGC |
| YTHDC1-R | GGAGGCACTACTTGATAGACGA |
| GAPDH-F | ACCACAGTCCATGCCATCAC |
| GAPDH-R | TCTAGACGGCAGG TCAGGTC |
